# A Review on Assessment and Treatment for Depression in Malaysia

**DOI:** 10.1155/2011/123642

**Published:** 2011-07-24

**Authors:** Firdaus Mukhtar, Tian P. S. Oei

**Affiliations:** ^1^Department of Psychiatry, Faculty Medicine and Health Sciences, Universiti Putra Malaysia, 43400 Serdang, Selangor, Malaysia; ^2^School of Psychology and CBT Unit, Toowong Private Hospital, University of Queensland, Brisbane, Queensland 4072, Australia

## Abstract

This paper aimed to review the literature on depression that focused on its assessment and treatment in Malaysia. PsycINFO, Medline, local journals were searched, and 18 published articles were included in this paper. Results indicate that research on depression in Malaysia, particularly validation studies and psychotherapy research, was weak and fragmented, with minimal empirical evidence available. Pharmacotherapy still dominated the treatment for depression, and, in terms of psychotherapy, Cognitive Behavioural Therapy (CBT) was recently practiced, but only a few studies have reported on the treatment efficacy of CBT. Major limitations of studies were noted, and, consequently, the problems that are associated with the implementation and future direction of clinical and research on depression in Malaysia were discussed. In short, the contribution of empirical research on the assessment and treatment for depression remained inconsistent and fragmented and urgently in need of further empirical investigation.

## 1. Introduction

It is projected that depression, an affective disturbance, will be among the leading causes of worldwide disability, by the year 2020 [1]. Across the Asia-Pacific region, rates of current or 1-month major depression ranged from 1.3 to 5.5% and rates of major depression in the previous year ranged from 1.7 to 6.7% [2]. Malaysia is no exception; in fact, depression is the most commonly reported mental illness in Malaysia. Depression is by far the most important and treatable condition and is projected to affect approximately 2.3 million Malaysians at some point in their lives [3]; yet depression remains underdetected and undertreated [4].

Theories of psychological disorders (particularly depression) are both clear and abundantly found in the literature. These theories can be broadly classified into either biological or psychosocial. Pharmacological theories of depression, such as amine dysregulations, are well established [5, 6] and thus provide a strong foundation for the pharmacological treatment of depression. It is clear that the efficacy of antidepressants, such as Selective Serotonin Reuptake Inhibitors (SSRI) and tricyclics, are well documented [7, 8]. Similarly, psychological theories such as Beck's cognitive theories are well articulated and generally accepted in the West [9–11]. 

It is also generally accepted in the literature that Cognitive Behaviour Therapy (CBT) is an effective way of treating depression [12–15]. 

In Malaysia, biological theories, and, thus, the pharmacological treatment of depression, are commonly used in clinical practices in community settings and hospitals; in fact, this is the main form of treatment for depression in Malaysia [16]. Unsurprisingly, the development of psychotropic medication in Malaysia has tended to ignore psychological aspects in the process of disease recognition and understanding, particularly for depression [4]. While psychotherapies for the treatment of depression are applied clinically in Malaysia, it is unfortunate that no empirical evidence to support such use has been established. Further, it is still unknown whether psychological instruments for the assessment of depression and the theories for depression are valid and reliable for use in Malaysia. 

Since psychological theories and, thus, treatment are more susceptible to cultural influences, it is therefore important to establish the validity of Western-derived psychological theories and psychological instruments for use in the treatment of depression in different cultures, such as Malaysia [17, 18]. Therefore, the aims of this paper were to review available articles related to the issues of assessment and treatment for depression.

## 2. Method

### 2.1. Selection of Studies

**Figure 1:**
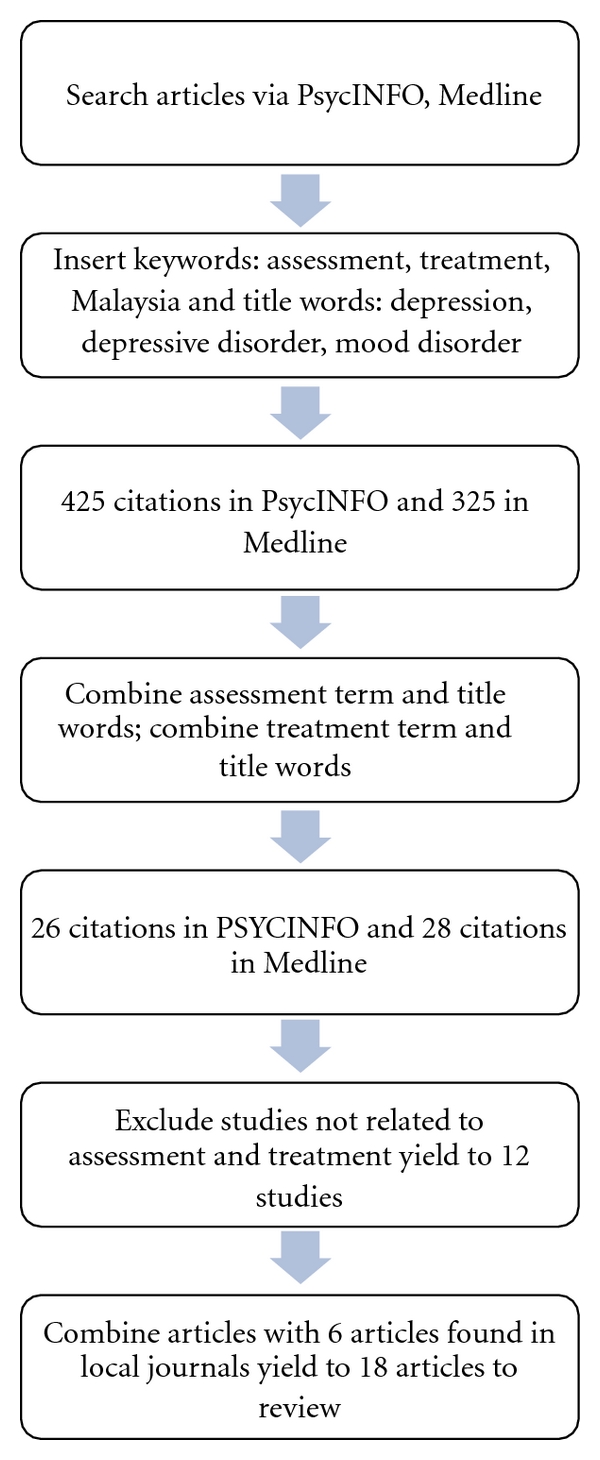
Flowchart of searching articles to review.

A search of the literature using electronic databases for PsycINFO and Medline (1970–present) was conducted (see Figure 1). Due to the scarcity of papers found in electronic databases, this paper also included manual searches of all available local journals in Malaysia, such as the Malaysian Journal of Psychiatry, the ASEAN Journal of Psychiatry, and the Journal of Malaysian Medical and Health Science, in order to meet the objective of this paper.

The search was refined to identify studies published in English over the last 30 years that included at least a cross-sectional and experimental study of depression using adult participants. Adult studies were targeted, in order to eliminate developmental differences in child or adolescent groups. Meta-analysis cannot be done due to the limited number of samples, variability of instruments used to measure depression, and insufficient reports on the statistical parts that are required for a systematic review. The publication years were chosen to incorporate the majority of studies, since the treatment for depression has been designed and researched.

Searches were conducted using the keywords, assessment, treatment, and Malaysia; and the following words in the title: depression, depressive disorder, and mood disorder. These keywords and title words were selected based on those found in the majority of papers collected earlier during the review process. Treatment keywords were combined to yield 425 citations in PsycINFO and 325 citations in Medline. The assessment terms were combined to produce 26 citations in PsycINFO and 28 citations in Medline. Studies were excluded for the following reasons: biological/physiological studies rather than treatment/assessment studies, depression was not the target of treatment, stress was misinterpreted as depression, and the article was a discussion of historical data rather than empirical studies. 

This review process cannot exclude papers with no information on effect size, as that would lead to a limited number of papers to review. This process resulted in 12 studies that were suitable for the review. An additional six studies taken from local journals, which met the selection criteria, were cited in these articles and added to the review, resulting in a total of 18 studies.

### 2.2. Description of Studies Reviewed

The assessment studies, arranged by their year of publication, are presented in Table 1 (a key of abbreviated terms is shown below the table). The following study features are summarised in Table 1: (a) study number and reference, (b) the target group, (c) the total number of participants, (d) representative of ethnicity, (e) gender, (f) measures used, (g) value of Cronbach's *α*, (h) validity study conducted, and (i) whether Exploratory Factor Analysis or Confirmatory Factor Analysis is conducted. The descriptor “target group” was included to investigate whether the assessment for depression was targeting patient or nonpatient samples. The sample size is important, since larger samples are known to give more reliable and representative results than small sample sizes (*n* < 30). The ethnicity and gender are important, because Malaysia is a multiethnic society; whereas, for gender, female is the most researched gender reported in studies worldwide. In order to investigate whether the instruments used to measure depression are validated with study on reliability and validity, the last three categories are importantly shown in the review. 

Meanwhile, for treatment studies, Table 2 summarizes all papers that were included in this review process. The following study features are summarised in Table 1: (a) study number and reference, (b) the sample and problem for treatment (depression symptoms or disorder), (c) ethnicity categories, (d) gender, (e) design of study, (f) intervention types, (g) the duration of intervention, (h) measures used, and (i) treatment outcome. The descriptor “problem for treatment” was included to investigate whether treatment was targeting patients with major versus minor depression, secondary depression, or depression symptoms. The sample size is important, since larger samples are known to give more reliable and representative results than small sample sizes (*n* < 30). Intervention types are important, in order to investigate dominant treatments sought or reported to overcome depression, whilst the duration and number of hours of intervention were included to give an understanding on the effectiveness of intervention related to time and cost. Finally, the type of outcome used to measure depression is important to investigate the reliability and validity of the tools used to assess depression and the treatment outcome.

## 3. Results

### 3.1. Description of Studies Included in the Review

A total of 18 studies fulfilled the above criteria for inclusion. Of these, seven studies were psychometric validation of a depression instrument and 11 studies were for treatment outcome of depression (Table 2). A total of 2,501 participants were represented, with sample sizes ranging from 1 (Studies 8, 12, and 15) to 1,050 (Studies 3, 4, and 6). A diversity of ethnicities were represented, where Malays were the dominant ethnicity involved in research (Studies 2, 3, 4, 6, 11, 12, 14, 15, and 18; only Malay subjects). The majority of studies (80%) involved participants who met research diagnostic criteria or DSM criteria for unipolar depressive disorders, while the remainder examined depression symptoms in participants drawn from at-risk groups in the community. In terms of gender, female subjects (*n* = 1279) participated the most in research for depression, but several studies did not report this category (Studies 1, 11, 13, 14, and 17).

### 3.2. Assessment Used to Measure Depression

In a review on assessments used to measure depression, three aspects will be discussed: subjects, measures used, and the statistical method. Table 1 shows validation studies on measures used for depression reported between 2001 and 2011. The range of subjects participated in these validation studies include students, general community, and patients (urological, primary care, psychiatric, diabetes, and infertility). The number of participants involved in these validation studies ranges from 237 to 1,050 subjects. Meanwhile, in terms of ethnicity, Malays form the majority that have been involved in validation studies in Malaysia. In terms of gender, females (*n* = 1185) and males (*n* = 569) have been investigated; however, gender was not reported in Study 1. 

In terms of measure, four studies (Studies 1, 3, 4, and 6) [19, 21–23] used the Beck Depression Inventory (BDI), followed by DASS-21 (Studies 5 and 7) [34, 35], and PHQ-9, HDRS and HADS (Study 2) [20]. Significantly, in Western studies, the BDI has been established as the most commonly used outcome measure either in a clinical setting or in research on depression [6]. Nevertheless, recent studies [22] support the notion that the BDI can be used as an instrument with confidence to measure levels of depression symptoms in Malaysians. Interestingly, two recent studies included cognitive measures (ATQ and DAS) for depression (Studies 3 and 6). These instruments are important because the cognitive behavioural approach emphasises alleviating negative cognitions in the treatment of depression.

Meanwhile, in terms of statistical analyses, both internal reliability and validity analyses were mentioned in all studies. Specifically, BDI studies have reported Cronbach's alpha between 0.56 to 0.90 and test-retest realibility (0.56–0.87). Meanwhile, for DASS-21, Cronbach's alpha and test-retest reliability are between 0.74–0.83 and 0.82–0.84, respectively. In addition, for the PHQ-9, HDRS and HADS, their Cronbach's alpha is 0.67 and test-retest reliability is 0.73. Meanwhile, Cronbach's *α* for ATQ is 0.90 and DAS is 0.82. For validity analyses, a combination of discriminate, concurrent, construct, and concurrent validities has been reported. Several studies also included specificity and sensitivity analysis to discriminate whether the tool is suitable to detect symptoms between medical and nonmedical populations. For an assessment to have robust and strong psychometric properties, Exploratory Factor Analysis and Confirmatory Factor Analysis are recommended, in order for a measure to be used as a valid tool. Now, only recent Studies 3, 4, 5, and 6 reported values on EFA and CFA, which has been shown to be at an acceptable range. 

To sum up this section, the above showed that two instruments for assessment of symptoms of depression had good valid and reliable psychometric information and thus can be used with some confidence for clinical research. Others such as PHQ-9, HDRS, and HADS have minimal psychometric validation information and thus must be used with caution.

### 3.3. Treatment Outcome of Depression in Malaysia

A review on treatment of depression that has been reported in Malaysia includes two general approaches (a) pharmacotherapy and (b) psychotherapy. 

Eight of the 12 studies used randomised controlled trial (RCT) type of research design, while three studies (Studies 8, 12, and 15) used case studies design. In terms of duration of treatment, all studies from 1976 to 2007 completed the intervention between 6 weeks and 17 months, but recent studies reported similar significant outcome completed the intervention in 4 weeks for 8 sessions of Group CBT approach. The majority of treatment studies (see Table 2) in Malaysia were using HDRS as outcome measure, but recent study (17) was using BDI instead. The only study that used cognition measures was Study 18 that emphasises on cognitive behavioural approach of assessment and intervention. 

In terms of subjects that have undergone treatment research of depression in Malaysia, both Malays and female are the dominant ethnic and gender that have been mostly researched. Only Studies 9, 10, and 16 were combining all three ethnic groups in Malaysia, otherwise other studies reported subjects and were only among Malays.

**(a) Pharmacotherapy**

Table 2 lists studies reported on the usage of pharmacotherapy intervention such as Amitriptyline (Studies 8, 9, and 14), Nomifensine (Study 8), Moclobemide (Study 10), Dothepin (12, 14, and 15), Maprotiline (12 and 14), Benzodiazepines (13), Imipramine (Study 14), Sertraline (16 and 17) Escitalaporam (17), and Fluoxetine (17).

Among all studies on pharmacotherapy, four studies (Studies 9, 10, 14, and 16) [25, 26, 30, 32] were using RCT method of research design. All four studies were using patients with depression, except Study 10 which included other diagnosis as well besides depression such as anxiety, bipolar, and schizophrenia. In terms of duration of treatment, most of the RCT studies for pharmacotherapy completed the study around the same time that range between six weeks and 9 weeks, and all four studies were using Hamilton Depression Rating Scale as an outcome measure before the Beck Depression Inventory and Depression Anxiety Stress Scale were validated in the country.

Besides RCT studies, interesting findings were from two case studies using two (Study 12) and one (Study 15) Malay patients with major depression who attributed their presenting symptoms to witchcraft or possession by evil spirits [28, 31]. These three patients were given dothiepin and maprotiline, respectively. Razali concluded from this report that, although patients were depressed before they went to see the *bomoh *(traditional healer), the specific psychosomatic symptoms developed after the *bomoh* convinced the patients that they had been charmed or their superstitious beliefs had been reinforced. They both improved slowly and were able to resume work 3 months and 6 weeks later.

Although Razali and Hasanah [16] stated that pharmacotherapy treatment for depression can be costeffective, there still remains an absence of any systematic treatment evaluation yet to conclude if the treatment is costeffective to all patients from both rural and urban regions.

**(b) Psychotherapies**

Besides pharmacological treatment, patients also have the alternative choice of psychotherapy treatments to treat symptoms of depression; several studies [13–16] support that a combination of pharmacotherapy and psychotherapy yields a better outcome.

In this paper, four types of psychotherapies have been reported such as psychodynamic (Study 8) [24], religious psychotherapy (Studies 11 and 13) [27, 29], supportive psychotherapy (Studies 11 and 13), individual cognitive behaviour therapy (Study 17) [36], and group cognitive behaviour therapy (Study 18) [33]. An item of significance in the studies by Azhar and Varma [27] and Razali et al. [29], Studies 11 and 13, which should be noted, is that the religious psychotherapy approach is based on cognitive behavioural intervention.

RCT study has been applied in four studies (Studies 11, 13, 17, and 18) for Malay patients with depression, except Study 18 which included patient with anxiety in their intervention. There are two RCT studies using religious psychotherapy, one is comparing to supportive psychotherapy and the other study was comparing to supportive psychotherapy and benzodiazepine. Meanwhile, for Cognitive Behaviour Therapy, Study 17 was comparing three groups of CBT with combination of Escitalopram, Sertraline, and Fluoxetine, respectively. The only Group CBT plus Treatment-As-Usual (TAU) study was conducted recently by comparing with Treatment-As-Usual group.

From all these studies, the recent study on Group CBT reported the shortest duration of treatment which is four weeks with eight sessions, and the outcome response is as successful as longer session. Otherwise, other psychotherapy studies in this review reported duration of treatment from 12 weeks to 26 weeks. However, Study 11 did not report on duration of sessions, but only reported that 15–20 sessions were completed by the participants.

This section also concluded that there is only one randomised control study on GCBT since its introduction in the early 1990s. Group intervention has been introduced in Malaysia in religious psychotherapy; thus, the GCBT study supports the notion that this form of treatment may be applicable to Malaysia, in particular the Malays as long as therapists are sensitive to the religious-sociocultural issues to which the group belongs. Hence, they concluded that the cognitive-behavioural approach complements and is compatible with the influence of religious values and has been especially effective in treating depressed patients [27]. The GCBT study is the first attempt to use a manual-based intervention in Malaysia, and response rates are encouraging.

Beside cognitive behaviour therapy, the efficacy of psychodynamic approach (Study 8) [24] cannot be concluded as it has been reported only in one study by using a single subject. Azhar and Varma [27] highlighted that the applicability of the psychodynamic approach for Malays is not inconsistent in Malaysia. Hatta [37] agreed that psychiatric services in Malaysia had their roots in the Western paradigm of medical practice, while Zain [38] warned that applicability of psychotherapy in Malaysia, in particular for Malay culture, could not readily accept the psychoanalytic model of Freud. Thus, Zain [38] argued that psychotherapy could be an effective treatment of depression in Malaysia, if carried out with a proper diagnosis and recruitment selection.

In terms of outcome measure, all RCT studies reported using Hamilton Depression Rating Scale, while Study 17 used Beck Depression Inventory and Hospital Anxiety and Depression Scale. Meanwhile, the GCBT study (Study 18), instead of using symptom outcome measure, reported using cognitive measures to assess the effectiveness of the treatment. Most of the studies did not report on follow-up sessions, clinical and statistical significance, and effect size except Study 18.

## 4. Discussion

This present paper aimed to review all studies performed in Malaysia, which were divided into two themes, namely, assessment and treatment of depression. It becomes clear that research on depression in Malaysia has a long way to go before psychiatric services are able to provide an ideal level of service for the community. 

Research studies vary in methodology and design and, thus, often present with conflicting findings in all areas including assessment and treatment. Consequently, small sample sizes, inadequate information on subjects' recruitment and randomisation of the sample, unclear diagnosis and treatment protocol procedure, lack of validated instruments, limited outcome measures or variables, problems with homogeneity of samples, and problems with generalisation of the results occur. In addition, for the treatment studies, information on effect sizes and also clinical significant change were seldom given.

For the psychometric assessment, it was unfortunate that only one study used exploratory and confirmatory factor analysis in order to validate the instruments used in Malaysia. Thus, weak statistical methodology used in validation studies overstated the reliability and validity of the instruments reviewed. While the BDI had been shown to have good psychometric properties, yet only Study 4 gave a precise numbers that used the BDI as a symptom measure for depression. It was noted in Tables 1 and 2 most used the HDRS and the HADS. Unfortunately, the psychometric properties of these measures for use in Malaysia are not solid. Furthermore, besides symptoms or mood change, cognitions have also been proven to be important variables for depression research. Only until recently that cognitive measures (Automatic Thoughts Questionnaire and Dysfunctional Attitude Scale) are validated and applied as a treatment outcome measures in the country. Since Malaysia is a multiculture society with 65% Malays/Bumiputera, 26% Chinese, and 7% Indians, the studies using Malay participants only may weaken the generalisability of the validity of the instrument for the whole of Malaysia.

## 5. Conclusions

In summary, there is evidence that some progress had been made in improving the assessment and treatment for patients with depression. Recently, an indigenously constructed and validated instrument for the screening of mental health problem was developed [39]. In terms of Group CBT, it has been growing favourable by many psychiatrist and primary care personnel because it can be delivered to a large number of participants with a minimum of time, thus making it an ideal intervention for research into, and treatment of, depression not just in psychiatric services but also in oncology, cardiac, and endocrine clinical services. It is hoped that, with further research and expansion of cognitive behavioural model application, patients will be able to receive evidence-based and contextually such as culturally and religiously appropriate treatments.

## Figures and Tables

**Table 1:** Studies on the assessment of depression in Malaysia.

Study	Target group	*n*	Ethnicity (%)	Gender (*n* or %)	Measure	Cronbach's (*α*)	Validity	EFA/CFA
(1) Quek et al. [19]	Urological	237	Malays Chinese (majority) Indian	NA	BDI	Internal Rel. (0.56–0.87); test-retest (0.56–0.87)	Discriminant; specificity and sensitivity	NA

(2) Azah et al. [20]	Primary care	265	Malays (100)	Male (*n* = 101) Female (*n* = 164)	PHQ-9, HDRS, HADS	Internal Rel. (0.67); test-retest (0.73)	Concurrent; specificity and sensitivity	NA

(3) Oei and Mukhtar [21]	Students, general community, primary care, and depressed patients	1050	Malays (100)	Male (*n* = 270) Female (*n* = 820)	ATQ, BDI, DAS	Internal Rel. (0.90)	Concurrent and discriminant; specificity and sensitivity	EFA/CFA

(4) Mukhtar and Oei [22]	Students, general community, primary care, and depressed patients	1050	Malays (100)	Male (*n* = 270) Female (*n* = 820)	BDI, ATQ, DAS	Internal Rel. (0.90)	Concurrent and discriminant; specificity and sensitivity	EFA/CFA

(5) Ramli et al.	Patients with diabetes	153	Malays (12) Chinese (16) Indian (17)	Male (75) Female (78)	DASS-21	Internal Rel. (0.74–0.79)	Construct	CFA

(6) Mukhtar and Oei [23]	Students, general community, primary care, and depressed patients (*n* = 113)	1050	Malays (100)	Male (*n* = 270) Female (*n* = 820)	DAS, BDI, ATQ	Internal Rel. (0.82)	Concurrent and discriminant; specificity and sensitivity	EFA/CFA

(7) Ramli et al.	Patients at Infertility Centre	246	Malays (230) Chinese (7) Indian (6) Others (3)	Male (123) Female (123)	DASS-21, HADS	Internal Rel. (0.81–0.83); test-retest (0.82–0.84)	Concurrent	NA

Note: Key to measure: (ATQ): Automatic Thoughts Questionnaire; (BDI): Beck Depression Inventory; (DAS): Dysfunctional Attitude Scale; (DASS-21): Depression Anxiety Stress Scale-21; (HARS): Hamilton Anxiety Rating Scale; (HDRS): Hamilton Depression Rating Scale; Hospital Anxiety and Depression Scale; (PHQ-9): Malay Version of Brief Patient Health Questionnaire; *n* = No. of subjects.

**Table 2:** Studies on the treatment of depression in Malaysia.

Study	Disorder or problem	Ethnicity (*n*)	Gender (*n*)	Design	Intervention	Duration of treatment	Outcome measure	Treatment result (+ve/−ve)
(8) Woon and Teoh [24]	Depression with hysterical personality disorder	Chinese (*n* = 1)	Female (*n* = 1)	Case study	Psychodynamic therapy and amitriptyline	1 year and 5 months	NA	Positive

(9) Ong and Lee [25]	Depressive neurosis and manic depressive	Malay (*n* = 9) Chinese (*n* = 6) Indian (*n* = 2)	Male (*n* = 6) Female (*n* = 11)	RCT	Nomifensine and amitriptyline	9 weeks	HDRS; Global clinical parameter	Positive with few adverse effects

(10) Indran [26]	Depression (12) Dysthymia (3) Anxiety (3) Bipolar (1) Schizophrenia (1) (Outpatient)	Malays (*n* = 4) Indian (*n* = 5) Chinese (*n* = 8) Others (*n* = 3)	Male (*n* = 9) Female (*n* = 11)	RCT	Moclobemide	6 weeks	HDRS; CGI; PGI	Positive with few adverse effects

(11) Azhar and Varma [27]	Depression	Malays (*n* = 64)	NA	RCT	Religious psychotherapy+ supportive therapy	15–20 sessions	HDRS	Positive

(12) Razali [28]	Depression	Malay (*n* = 2)	Male (*n* = 1) Female (*n* = 1)	Case study	Dothiepin and maprotiline	3 month and 1 month	NA	Positive

(13) Razali et al. [29]	Depression (*n* = 100) and anxiety (*n* = 103)	Malays (*n* = 203)	NA	RCT	Religious psychotherapy+ supportive therapy + benzodiazepines or antidepressants	26 weeks	HARS; HDRS	Positive

(14) Razali and Hasanah [30]	Depression (Outpatient)	Malay (*n* = 82)	NA	RCT	Amitriptyline, imipramine, dothiepin and maprotiline	8 weeks	HDRS	Positive

(15) Razali [31]	Masked Depression (Outpatient)	Malay (*n* = 1)	Female (*n* = 1)	Case Study	Dothiepin	6 weeks	NA	Positive

(16) Ng and Stevens [32]	Depression	Malaysian Chinese (*n* = 13); others (*n* = 32)	Male (*n* = 6) Female (*n* = 8)	RCT	Sertraline (SSRI)	6 weeks	HDRS; CGI; LUNSERS; Plasma measurement	Positive with few adverse effects

(17) Azhar et al.	Depression (Outpatient)	*n* = 96	NA	RCT	CBT + ESCCBT + STRCBT + FXT	12 weeks	HADS; BDI; WHO-QOL	Positive; CBT + ESC show better

(18) Mukhtar et al. [33]	Depression (Outpatient)	Malays (*n* = 113)	Male (*n* = 51) Female (*n* = 62)	RCT	GCBT + TAUTAU	8 sessions; 1 month	ATQ; DAS	Positive and maintained at 3 and 6 month followups

Note: KEY to intervention (alphabetical order): Group Cognitive Behaviour Therapy (GCBT); Selective Serotonin Reuptake Inhibitor (SSRI); Escitalopram (ESC); Sertraline (STR); Fluoxetine (FXT); Treatment-as-Usual (TAU)Note: KEY to measures (alphabetical order): Automatic Thoughts Questionnaire (ATQ); Beck Depression Inventory (BDI); Clinical Global Impression (CGI); Dysfunctional Attitude Scale (DAS); Hamilton Anxiety Rating Scale (HARS); Hamilton Depression Rating Scale (HDRS); Liverpool University Neuroleptic Side-effect Rating Scale (LUNSERS); Patients Global Improvement (PGI).
